# Investigation of ciguatoxins in invasive lionfish from the greater caribbean region: Implications for fishery development

**DOI:** 10.1371/journal.pone.0198358

**Published:** 2018-06-20

**Authors:** D. Ransom Hardison, William C. Holland, H. Taiana Darius, Mireille Chinain, Patricia A. Tester, Damian Shea, Alex K. Bogdanoff, James A. Morris, Harold A. Flores Quintana, Christopher R. Loeffler, Dayne Buddo, R. Wayne Litaker

**Affiliations:** 1 National Oceanic and Atmospheric Administration, Center for Coastal Fisheries and Habitat Research, Beaufort, North Carolina, United States of America; 2 Institut Louis Malardé (ILM)-UMR 241 EIO, Laboratory of Toxic-Microalgae, Papeete, Tahiti, French Polynesia; 3 Ocean Tester, LLC, Beaufort, North Carolina, United States of America; 4 North Carolina State University, Environmental Chemistry and Toxicology Laboratory, Raleigh, North Carolina, United States of America; 5 U.S. Food and Drug Administration, Division of Seafood Science and Technology, Gulf Coast Seafood Laboratory, Dauphin Island, Alabama, United States of America; 6 University of the West Indies, Discovery Bay Marine Laboratory and Field Station, Discovery Bay, St. Ann, Jamaica WI; Department of Agriculture and Water Resources, AUSTRALIA

## Abstract

Lionfish, native to reef ecosystems of the tropical and sub-tropical Indo-Pacific, were introduced to Florida waters in the 1980s, and have spread rapidly throughout the northwestern Atlantic, Caribbean Sea and the Gulf of Mexico. These invasive, carnivorous fish significantly reduce other fish and benthic invertebrate biomass, fish recruitment, and species richness in reef ecosystems. Fisheries resource managers have proposed the establishment of a commercial fishery to reduce lionfish populations and mitigate adverse effects on reef communities. The potential for a commercial fishery for lionfish is the primary reason to identify locations where lionfish accumulate sufficient amounts of ciguatoxin (CTX) to cause ciguatera fish poisoning (CFP), the leading cause of non-bacterial seafood poisoning associated with fish consumption. To address this issue, an initial geographic assessment of CTX toxicity in lionfish from the Caribbean and Gulf of Mexico was conducted. Lionfish samples (n = 293) were collected by spearfishing from 13 locations (74 sampling sites) around the Caribbean and Gulf of Mexico between 2012 and 2015. The highest frequencies of lionfish containing measurable CTX occurred in areas known to be high-risk regions for CFP in the central to eastern Caribbean (e.g., 53% British Virgin Islands and 5% Florida Keys). Though measurable CTX was found in some locations, the majority of the samples (99.3%) contained CTX concentrations below the United States Food and Drug Administration guidance level of 0.1 ppb Caribbean ciguatoxin-1 (C-CTX-1) equivalents (eq.). Only 0.7% of lionfish tested contained more than 0.1 ppb C-CTX-1 eq. As of 2018, there has been one suspected case of CFP from eating lionfish. Given this finding, current risk reduction techniques used to manage CTX accumulating fish are discussed.

## Introduction

Invasive lionfish (*Pterois volitans* and *P*. *miles*) were first observed in U.S. waters off the Florida coast in 1985. Sightings remained sporadic in southeast Florida until 1999 and 2000 when individual lionfish were reported off Bermuda, North Carolina, South Carolina, and Georgia. By 2001, lionfish were considered to be established in the northwestern Atlantic [1]. Due to their high reproductive capabilities and lack of natural predators, lionfish rapidly expanded their range and have become one of the most abundant top level predators throughout the U.S. South Atlantic Bight, Caribbean, and Gulf of Mexico [2]. Stomach content analysis has confirmed their diet includes over 50 families of fish and invertebrate prey. The dominant prey items consumed are teleost fish followed by shrimp and other crustaceans [3]. Laboratory [4, 5] and field studies [6, 7] estimate average daily consumption rates for lionfish exceed 0.089 g prey g lionfish^-1^, with specific rates varying in proportion to the size of the lionfish and water temperature. These consumption rates can be combined with lionfish size and density data to estimate annual lionfish prey consumption at a given population level. For example, an average population of 393 lionfish hectare^-1^ can consume ~490,000 prey items hectare^-1^ year^-1^, or about 930 kg of prey, when feeding at 60% of maximum consumption rates [4]. These high rates of predation have been shown to significantly reduce benthic invertebrate biomass [8], as well as fish recruitment [9], fish biomass [10], and species richness [11] in tropical and subtropical reef systems.

Since lionfish have no natural predators in the Caribbean and Atlantic basins to help control populations, and they have high reproductive potential, researchers agree that lionfish cannot be eradicated from the Atlantic [2]. However, local scale efforts are being made in an attempt to minimize their negative impacts on the reefs through organized and sustained removal efforts (e.g., recreational lionfish fishing derbies and tournaments). As part of a campaign to stimulate large-scale control, many locations have begun promoting the commercialization of lionfish, particularly as a food fish. Lionfish flesh is firm, white, flaky, and higher in healthy omega-3 fatty acids than many other commonly consumed species [12]. Lionfish have become a widely popular seafood item, especially as an ornate, luxury, and ‘green’ meal. They are now being harvested recreationally and commercially throughout most of the invaded regions and are served in over 160 restaurants [13]. Between 2011 and 2014, 23,324 kg of lionfish was commercially harvested from U.S. federal waters alone and this number is likely an underestimate due to a lack of reporting [14]. These landings had an estimated total dockside value of $222,897 USD, with prices ranging from $9.35 to $10.48 per kg [14].

Population models that were developed to estimate the exploitation rates required to eradicate lionfish can provide insight into the sustainability of lionfish as a fishery resource. Predicted annual exploitation rates between 35 and 65% of the total population would be required to eliminate lionfish [15], and a predicted monthly exploitation of 27% of the adult population would result in zero net growth [16]. The level of resources needed to sustain these removal rates has not been explored. Lionfish are highly fecund (~2 million eggs per female per year), abundant (over 500 per hectare in some locations), and widely distributed (nearly all marine habitats from 0 m to 300 m depth) throughout the invaded regions making them uniquely capable of having wide-reaching impacts without human intervention. Without major advancements in lionfish fishing practices, it is unlikely these exploitation rates will be achieved in the near future. The development of a lionfish fishery not only has the potential to impart regional control and mitigation of ecological impacts, but it could also represent an unexploited economic opportunity for fishing communities.

An important concern with developing a fishery for lionfish is they may contain toxins that could result in ciguatera fish poisoning (CFP) [2, 17, 18]. CFP is caused by consuming fish that have accumulated lipid-soluble ciguatoxins (CTX) derived from precursor toxins produced by microalgae of the genus *Gambierdiscus*. Initial CFP symptoms include gastrointestinal distress characterized by nausea, vomiting, abdominal pain, and diarrhea [19]. Other acute cardiovascular symptoms typically include hypotension and bradycardia. Chronic symptoms are generally neurological and manifest as paresthesia, muscle and joint pain, fatigue, and cold allodynia (hot/cold temperature sensation reversal) [19]. While infrequent, CFP related deaths have been reported from Cuba (n = 1), Dominica (n = 2), Mexico (n = 2), Hawaii (n = 2), Puerto Rico (n = 3), and Venezuela (reported as several deaths) [19]. CFP cases are vastly underreported, but with an estimated 50,000 cases per year, it is still the most common non-bacterial illness associated with eating fish [20]. CFP intoxication is endemic to the tropics-subtropics, but is not limited to these areas due to the global import of potentially ciguatoxic fish.

Ciguatoxins are found in fish from the Pacific and Indian oceans, as well as the Caribbean. These toxins are classified by their region of isolation, resulting in P-CTX (Pacific-ciguatoxin), C-CTX (Caribbean-ciguatoxin), and I-CTX (Indian-ciguatoxin), with each toxin structure being different. These structural differences result in P-CTXs being approximately 10-fold more potent than C-CTXs [21]. Consequently, a guidance level for CTXs in fish has been set at 0.1 ppb C-CTX-1 eq. and 0.01 ppb for P-CTX-1 eq. by the U.S. Food and Drug Administration (FDA) [22]. These guidance levels were set based on a 10-fold reduction of the lowest concentration of CTXs found in meal remnants known to cause human illnesses [22].

Two recent, regional studies have documented CTXs in lionfish from the eastern Caribbean. One study [17] sampled 153 lionfish from the U.S. Virgin Islands and detected CTXs in 40% of the samples with 12% above the FDA guidance level of 0.1 ppb C-CTX-1 eq. Another investigation [18] sampled 120 lionfish from the French Antilles and detected CTXs in 23% of the samples with 18% above the FDA guidance level. These studies indicate that promoting the consumption of invasive lionfish can be a challenging issue for resource managers, given the inherent variability of CTX concentrations above recommended guidelines among and within locations. Before proceeding with the promotion of harvesting lionfish, important questions need to be addressed, such as: 1) Are lionfish a safer alternative to other locally consumed reef fish with regard to CTXs; and 2) From what locations are lionfish likely to be ciguatoxic, and can these higher risk areas be reasonably avoided? The goal of this study was to provide the public, including fishers, consumers, and resource managers from around the region with baseline information on the frequency and toxicity of CTXs in invasive lionfish on a broad geographic scale.

## Methods

The research was conducted by the National Oceanic and Atmospheric Administration (NOAA) at a NOAA facility. No institutional animal care and use committee exists within NOAA to review research protocols. NOAA does, however, adhere to all animal care and use regulations and policies outlined in documents such as the Guide for the Care and Use of Laboratory Animals [23], the Animal Welfare Act and Animal Welfare Regulations.

**Fig 1:**
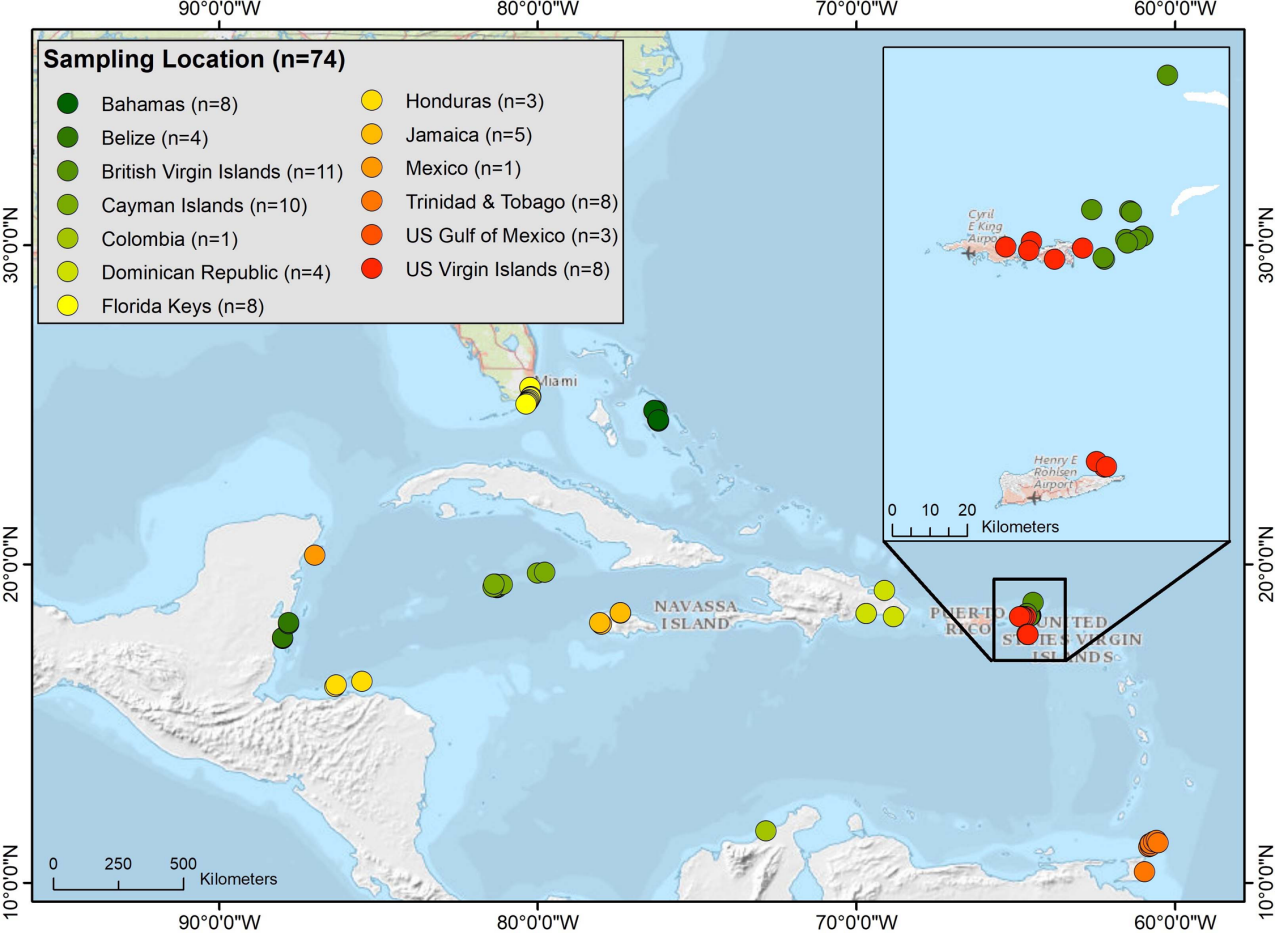
Map showing distribution of sampling sites where fish were collected. Sample sites located within each location are identified as “n”.

Coastal managers from each location were asked to provide a minimum of fifteen lionfish from areas (marine sanctuaries, state parks, etc.) they manage. The lionfish were collected from random multiple sites within each location to achieve a representative sample population. These efforts resulted in frozen lionfish (n = 293) being provided from spearfishing efforts from 13 locations (74 sampling sites) around the Caribbean and Gulf of Mexico between 2012 and 2015 (Fig 1 and Table 1). All fish were collected by researchers and managers using pole spears and were placed on ice immediately after capture. This method is the fastest, safest, and most humane method for capturing venomous lionfish in the field [2]. Time, location, depth, habitat type, and fish length and weight were recorded for each sample. The fish weighed on average 431 g (median = 377 g) and were collected at depths ranging from 5–47 meters. Although lionfish were sampled from multiple sites in each location, results were pooled to represent lionfish ciguatoxicity for each general area. The sampling locations and the number of fish sampled in parentheses were as follows: Bahamas (15), Belize (15), British Virgin Islands (15), Cayman Islands (15), Colombia (15), Dominican Republic (15), Florida Keys (20), Flower Garden Banks National Marine Sanctuary (33), Honduras (15), Jamaica (29), Mexico (15), Trinidad and Tobago (76), and the U.S. Virgin Islands (15) (Fig 1). Samples were shipped or personally transported to the NOAA Beaufort Laboratory, Beaufort, NC in coolers with dry ice and/or freezer packs. All samples were frozen (–20°C) immediately after capture and remained frozen during transport pending toxin extraction.

**Table 1:** Location based fluorescent receptor binding assay (RBA_F_) results list the date of sampling, number of lionfish screened and the number of positive samples. Following the RBA_F_ screening, all the positive samples (n = 30) plus a subset of negative samples (n = 24) were next analyzed using CBA-N2a and LC-MS/MS. The negative samples identified by RBA_F_ were also negative when measured by CBA-N2a and LC-MS/MS. CBA-N2a and LC-MS/MS confirmed that a subset of the samples screened contained measurable levels of C-CTX-1. Location abbreviations FGBNMS and USVI stand for Flower Garden Banks National Marine Sanctuary and United States Virgin Islands, respectively.

		RBA_F_		CBA-N2a and LC-MS/MS		
Area sampled	Date collected	Total fish analyzed	# of CTX-like positive fish	Total fish analyzed	# of CTX-like positive fish	# confirmed by LC-MS/MS
Bahamas	October 2013	15	0	5	0	0
Belize	August, October 2013	15	3	3	0	0
British Virgin Islands	July, August 2013	15	9	11	8	6
Cayman	October 2013	15	0	0	0	0
Colombia	September 2013	15	0	5	0	0
Dominican Republic	September, October 2013	15	2	5	0	0
Florida Keys	September, December 2014; January, February 2015	20	5	5	2	1
FGBNMS	August, September, November 2015	33	0	0	0	0
Honduras	October 2013	15	1	1	0	0
Jamaica	July 2012	29	1	4	0	0
Mexico	August 2013	15	1	1	0	0
Trinidad and Tobago	April, May 2015	76	6	11	0	0
USVI	May, June 2013	15	2	3	0	0
**Total survey**	**June 2012 –November 2015**	**293**	**30**	**54**	**10**	**7**

Lionfish (n = 293) were initially screened for CTX activity by a fluorescent receptor binding assay (RBA_F_). Samples positive for CTX-like activity via the RBA_F_ were subsequently sent to the FDA Gulf Coast Seafood Laboratory, Dauphin Island, AL, USA to be further analyzed by a neuroblastoma cell based cytotoxicity assay (CBA-N2a) and confirmed for the presence of C-CTX-1 by liquid chromatography tandem mass spectrometry (LC-MS/MS).

### Toxin extraction for RBA_F_

Fish tissue samples were extracted using the protocol described in Darius et al. [24]. Briefly, specimens were thawed to room temperature and triplicate 5 g subsamples of flesh were removed, placed in a 50 mL conical falcon tube, and heated to 70°C for at least one hour. This step eliminated native fluorescence in the samples, which can interfere with signal to noise ratios of the RBA_F_, and eliminates the possibility of detecting false positives by denaturing any remnant lionfish venom in the flesh [25]. Ciguatoxins were extracted from each tissue sample with 7 mL of methanol by homogenizing with a finger sonicator (Q-Sonica, Q700, Newtown, Connecticut) for 1 minute. The tubes containing tissue and methanol were capped and placed in a water bath sonicator (Branson, 1800, Danbury, Connecticut) for two hours and then incubated at room temperature overnight (14–16 h). The extracts were then centrifuged at 4700 rpm for 5 minutes. The resulting supernatant was decanted into a 20 mL glass scintillation vial and adjusted to 70% methanol: 30% water (typically this only required adding ~3 mL of Milli-Q water). Each supernatant was then passed through Waters Sep Pak^®^ Plus C_18_ solid phase extraction (SPE) columns (WAT020515, 360 mg) preconditioned with 10 mL of 70% methanol: 30% water using a Supelco Visiprep™ DL vacuum manifold (Sigma Aldrich, St. Louis, Missouri). After extracts were loaded onto SPE columns, they were washed with 70% methanol: 30% water twice (7 mL x 2) and eluted with 7 mL of 90% methanol: 10% water into a glass scintillation vial. Vials were then transferred to a nitrogen evaporator (Organomation Associates, Inc., N-EVAP 111, Berlin, Massachusetts) and volumes were reduced to less than 2 mL under ultra-high purity nitrogen at 50°C. The concentrated extracts were transferred to 2 mL glass high performance liquid chromatography vials and blown to dryness with nitrogen, sealed, and stored at ‒20 °C until analyzed. Prior to running an assay, the dried extracts were resuspended in RBA_F_ buffer.

### RBA_F_ assay protocol

Following extraction, lionfish samples were screened using the RBA_F_ assay to examine lionfish extracts for the presence of CTX-like activity [26–28]. The RBA_F_ positive (n = 30), as well as negatives (n = 24), were later screened by the FDA Gulf Coast Seafood Laboratory using the CBA-N2a.

The kits containing synaptosomes and fluorescently labeled brevetoxin (BODIPY^®^- PbTx-2) needed to perform the RBA_F_ can be purchased from SeaTox Research, Inc. at MARBIONC, Wilmington, NC, USA. The BODIPY^®^- PbTx-2, which competes with CTXs in the sample for the synaptosome sodium channel sites, was dissolved in 200 proof ethanol to produce a 0.1 mM solution and stored at ‒20°C in the dark.

The assay procedure has been previously described in detail [26]. Briefly, a 96-well plate was read using a FLUOstar Omega fluorometer (BMG Labtech, Germany) with a 505 nm long band pass dichroic, a 490–10 nm excitation filter, and a 520–10 nm emission filter to obtain the relative fluorescence units (RFUs) for each well. The lowest value RFU for the Pacific ciguatoxin-3C (P-CTX-3C) standard curve (highest toxin concentration) was subtracted from each well to account for any background fluorescence due to non-specific binding of the BODIPY^®^- PbTx-2 to the synaptosomes or filter plate. The data were then scaled so that the corrected RFUs from the standard curve samples containing no P-CTX-3C represented 100% specific binding and the RFUs from the highest P-CTX-3C concentration was set to 0% binding. The normalized RFUs versus concentration data were fitted to a 4-parameter logistic model = 4PL, Hill Slope Model) to estimate IC_50_ values using Graphpad software (GraphPad Prism version 6 for Windows, GraphPad Software, San Diego, California, USA, www.graphpad.com). The time required for the RBA_F_ to reach equilibrium was found to be optimum at 1.5 h [26]. Because the lionfish CTX levels in this study were low, a linear standard curve ranging from 0.1–1.0 ppb P-CTX-3C eq. was used. These standard curves were highly replicable [26].

P-CTX-3C (WAKO Chemicals, Richmond, Virginia, USA) was used for constructing standard curves because it is one of the few congeners currently commercially available in sufficient quantity. C-CTX-1, and its epimer C-CTX-2, which are the dominant CTX congeners found in Caribbean fish, would be the preferred standard, but are only available in minute quantities.

Extraction recovery efficiencies (83±2%) were estimated by spiking fish tissues with P-CTX-3C, extracting as described above, and calculating the resulting concentration from the linear standard curve. Certainly, P-CTX-3C may not extract with the same efficiency as C-CTX-1. However, until sufficient quantities of C-CTX-1 are available, the precise extraction efficiency of C-CTXs remains unknown and P-CTX-3C standards will have to serve as a proxy.

### Extraction protocol for the neuroblastoma 2A assay and LC-MS/MS

The lionfish samples that were positive (n = 30) (>0.05 ppb P-CTX-3C), as well as negatives (n = 24), by the RBA_F_ assay were shipped to the FDA for testing using the CBA-N2a assay and confirmation by LC-MS/MS. Those samples were extracted according to Dickey [29]. Briefly, an acetone extraction at 2 mL/g tissue homogenate was initiated. Acetone extracts were chilled at ‒20°C for 12 h and the precipitate was removed by filtration. The supernatant was dried and the residue was partitioned between 80% methanol and 95% *n*-hexanes. The hexane wash was discarded and the methanol fraction was dried by nitrogen. Residues were then dissolved in water and extracted with chloroform three times. The chloroform phases were then dried by nitrogen. Sample cleanup was done with silica and aminopropyl (NH_2_) solid phase extraction (SPE) cartridges before analysis by CBA-N2a and LC-MS/MS.

### Neuroblastoma 2A cell based cytotoxicity assay

The CBA-N2a assay was run according to published protocols for screening fish extracts [29–32]. Briefly, the neuroblastoma-2a cell line (N2a) was obtained from the American Type Culture Collection (ATCC CCL 131) and grown at 37°C, humidified 5% CO_2_ atmosphere in RPMI media. To prepare for toxicity analysis, N2a cells were harvested with a trypsin-ethylenediaminetetraacetic acid solution and seeded into each well of a 96-well microtiter plate at 40,000 cells per 200 μL of growth medium using the same growth conditions.

Plates seeded with N2a cells were incubated for 24 h until each well was >90% confluent. The standards, controls, and samples were then added and incubated for 20–24 h. Controls, standards, and sample dilutions were assayed in triplicate wells on plates. Full dose response curves (8-dilutions) of C-CTX-1 standard and sample extracts were prepared with sensitized (+Ouabain octahydrate 250 μM and Veratridine hydrochloride 25μM) and non-sensitized (-Ouabain octahydrate and Veratridine hydrochloride) cells. Sensitized cells were used to determine the lethal concentration of extract required to kill 50% of the tested cells in culture (LC_50_) and compared with a C-CTX-1 standard. C-CTX-1 standard curve ranged from 0.019–2.5 pg/well. Cell viability was assessed after 20–24 h of toxin exposure at 37°C using the colorimetric 3-(4,5-dimethylthiazol-2-yl)-2,5-diphenyl tetrazolium bromide assay [29]. Results were expressed as ppb C-CTX-1 eq. wet-weight. Data analysis for CBA-N2a results was performed using JMP software (v9, SAS Institute, Cary NC, USA).

### LC-MS/MS confirmation

Qualitative confirmation of C-CTX-1 was carried out using a modified protocol [18, 26]. Analysis was performed using an Agilent 1260 LC system (Agilent Inc., Palo Alto, California) coupled to a QTRAP 4000 mass spectrometer (Applied Biosystems, Inc., Foster City, California). Elution of analytes was performed on a Kinetex C8 (75 x 2.1 mm; 2.6 μm) column (Phenomenex, Torrance, California) using water and 95% aqueous acetonitrile with both containing 0.1% formic acid. LC-MS/MS settings have been reported previously [26]. Ions monitored for qualitative confirmation of C-CTX-1 were the dehydrated C-CTX-1 ion (M + H‒H_2_O)^+^ as a precursor for the following MRM ion transitions: *m/z* 1123.6 > 1105.6, 1123.6 > 1087.6, 1123.6 > 1069.6.

## Results

The initial screening results using the RBA_F_ assay showed approximately ten percent (n = 30) of the total lionfish exhibited CTX-like activity (Table 1). All RBA_F_ positive samples and 24 randomly selected negative samples were subsequently analyzed at the FDA Dauphin Island laboratory using the CBA-N2a assay and LC-MS/MS. A comparison of duplicate RBA_F_ and CBA-N2a sample results showed that (1) CBA-N2a presented CTX-like activity in some but not all of RBA_F_ positive samples, (2) that none of the negative samples identified by RBA_F_ contained more than trace amounts of CTX (<0.01 ppb C-CTX-1 eq.) when measured by the CBA-N2a assay–i.e. 99.3% of the total lionfish had concentrations below the 0.1 ppb C-CTX-1 eq. FDA recommended guidance levels, and (3) the RBA_F_ consistently overestimated the CBA-N2a results. The conservative nature of the RBA_F_ supports the method as a valuable, rapid screening tool with any positive samples needing to be further analyzed by CBA-N2a and/or LC-MS/MS [26, 33].

**Fig 2:**
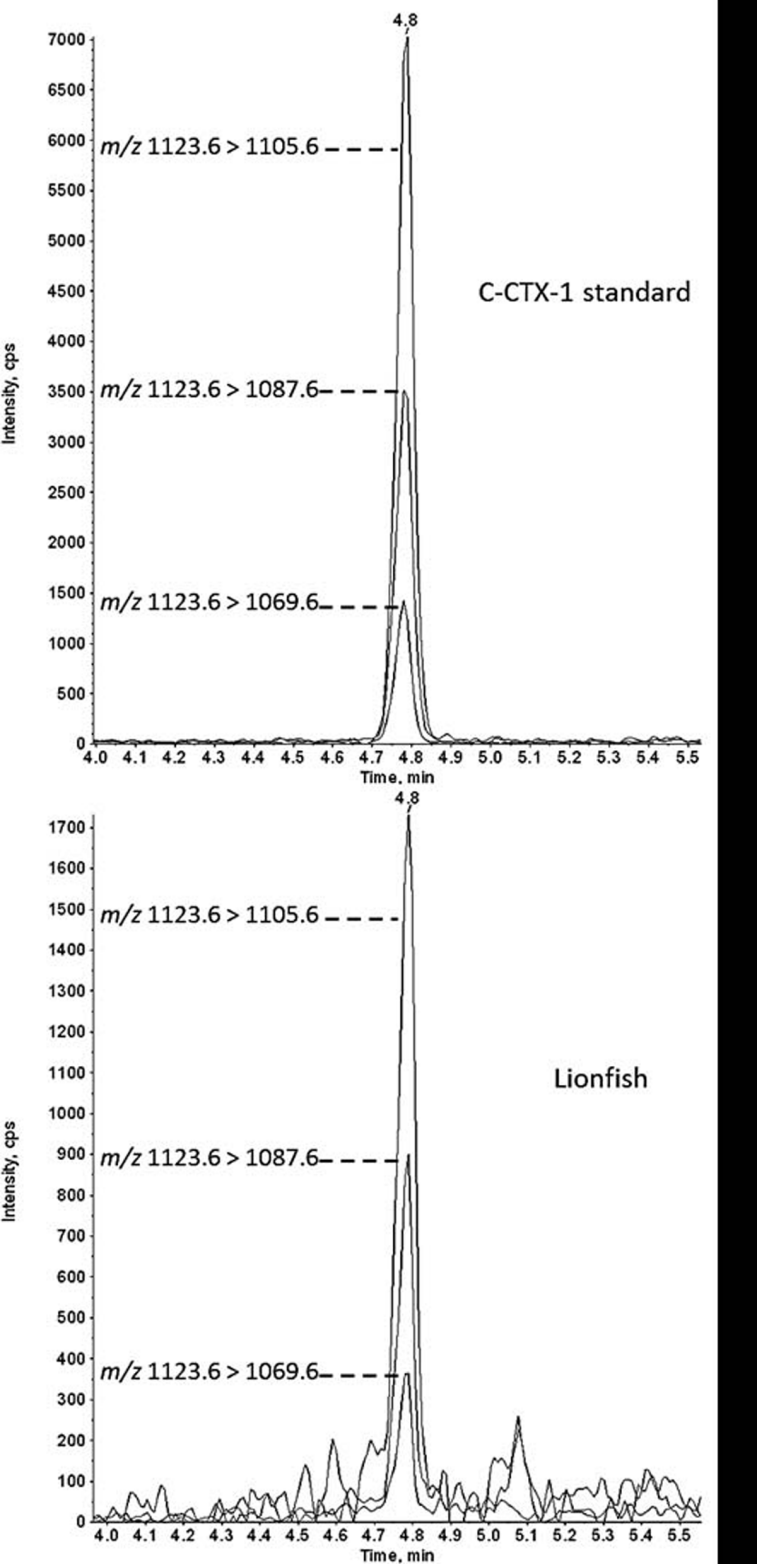
LC-MS/MS chromatogram for confirmation of C-CTX-1. The chromatogram shows the three characteristic confirmatory ion transitions (*m/z* 1123.6 > 1105.6, 1123.6 > 1087.6, and 1123.6 > 1069.9) and the retention time (4.8 min) of a C-CTX-1 standard and in lionfish.

**Fig 3:**
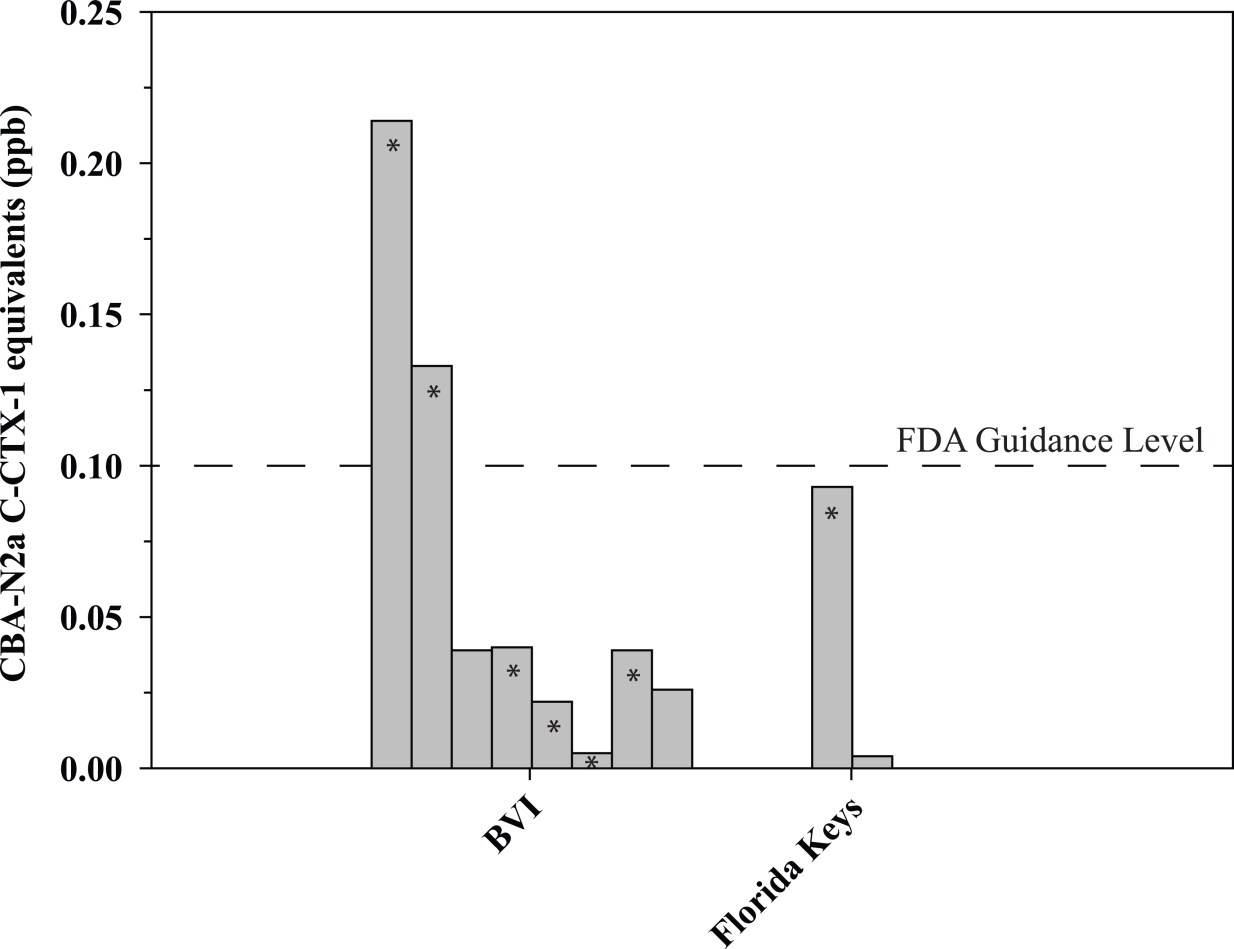
The CBA-N2a detectable CTX concentrations in lionfish from two of the sampling locations are shown as C-CTX-1 eq. values in ppb. The number of fish analyzed at each location was 11 for the British Virgin Islands (BVI) and 5 for the Florida Keys. C-CTX-1 was confirmed by LC-MS/MS (denoted by an *) in six of the samples from BVI and one from Florida Keys. The horizontal dashed line indicates the FDA guidance level of 0.1 ppb for C-CTX-1 eq.

The CBA-N2a results showed a prevalence of 3.4% of the total lionfish contained CTX while the LC-MS/MS (Fig 2) confirmed the presence of C-CTX-1 in 2.4% of the same sample set and the results indicated 0.7% (n = 2) of the total lionfish examined contained CTX concentrations above the FDA guidance level (Tables 1 and 2). Those concentrations were 0.133 and 0.214 ppb C-CTX-1 eq. and both fish were from the British Virgin Islands (Fig 3). The highest frequency of CBA-N2a CTX positive fish (53%) occurred in the British Virgin Islands, with only one positive sample found in the Florida Keys and no confirmed C-CTX-1 in lionfish from the remaining 11 areas.

**Table 2:** Ciguatoxin concentrations in lionfish reported from two previous studies done in the Lesser Antilles and US Virgin Islands determined by CBA-N2a compared to the CTX estimates found in this study. ND = CTX activity not detected.

Study	Location	N	Fish containing measurable CTX (%)	Fish containing CTX concentrations above FDA guidance level (%)
This study	Caribbean & Gulf of Mexico	293	2.4	0.7
[17]	US Virgin Islands	153	40	12
[18]	St. Barthélemy	55	49	40
[18]	Guadeloupe	60	ND	ND
[18]	St. Martin	5	ND	ND

## Discussion

This study represents the first broad geographic assessment of CTX concentrations in lionfish from the Caribbean and Gulf of Mexico. Sampling sites ranged from Mexico and Belize in the western Caribbean to the Bahamas and British Virgin Islands in the eastern Caribbean and from the Flower Garden Banks National Marine Sanctuary in the northern Gulf of Mexico to Trinidad and Tobago in the southern Caribbean (Fig 1; Table 1). Over this geographic range only 0.7% of the 293 lionfish tested (13 locations), exceeded the FDA guidance level of 0.1 ppb C-CTX-1 eq. (Fig 2; Tables 1 and 2) [22]. Only one sample exceeding the guidance level contained more than 0.2 ppb C-CTX-1 eq. Aggregating the data, however, obscures the large amount of inter site variability in toxicity that exists. At a local level, the percentage of fish containing measurable CTX ranged from 0 to 53% (Fig 3; Table 1). The 53% estimate was from a hot spot in the British Virgin Islands known for having higher than average CFP incidence rates [34]. Similarly, the US Virgin Islands are another hot spot, as indicated by a recent study by Robertson et al. [17], showing 40% of the lionfish (n = 153) tested contained detectable CTX with 12% above the FDA guidance level (Table 2). In contrast, our study did not find detectable CTX in the lionfish tested from this location. A potential explanation for this is the fine-scale differences in habitat suitability among sampling locations. The study by Olsen et al. [34] found CFP risk in the US Virgin Islands was high for fish caught on the south side of St. Thomas and St. John, low for those from the north side of St. Thomas and St. John, and low to moderate for fish from the northeast and southern costs of St. Croix. A majority of the fish sampled by Robertson et al. [17] were from the high CFP area located on south side of St. Thomas, which is also where the fish containing the highest CTX concentration in the study was collected. In contrast, all but one of the fish from the US Virgin Islands surveyed in this study were taken from the north side of St. Thomas or from St. Croix where CFP risk is generally lower [34]. Similar spatial variation was also observed in the French Antilles [18]. Forty-nine percent of the samples (n = 55) from Saint Barthélemy contained measurable CTXs, with 40% exceeding the FDA guidance level. In contrast, lionfish from the nearby islands of Guadeloupe (n = 60) and Saint Martin (n = 5) did not contain measurable CTXs [18]. These results indicate that in areas where other fish are known to be ciguatoxic, lionfish will also accumulate CTXs, and the degree of accumulation is habitat specific. Areas prone to CFP tend to be concentrated in the central and eastern Caribbean, (Fig 1; Tables 1 and 2) [18, 35, 36]. In contrast, the most complete survey of a low CFP region was done on Trinidad and Tobago (Table 1). A total of 76 fish from geographically dispersed local habitats were sampled and none contained measurable CTX.

One reason for the variation of the CTX found in fish from adjacent locations may be the species and abundance of the *Gambierdiscus* spp. present. It is known that *Gambierdiscus* spp. distribution and abundance are influenced by local water temperatures (optimum range between 21 and 31°C), relatively high, stable salinities (>33) and habitat characteristics including abundant macrophytes and algal turfs or substrates to which cells can attach, low to moderate turbulence/flow rates (leeward side many islands), and water depth <100 m [35, 36]. Nutrients are not considered a limiting factor for *Gambierdiscus* spp. because they have direct contact with the sediment layer or macrophyte substrate where elevated nutrient levels occur [37, 38]. *Gambierdiscus* cells also do poorly in areas receiving direct runoff from land masses [39]. Logically, habitats that foster large *Gambierdiscus* populations would produce the most toxic fish if all *Gambierdiscus* species are equally toxic. However, studies show that small *Gambierdiscus* blooms are sometimes more toxic than larger ones, implying that species-specific differences in toxicity exist [40]. This is supported by a recent study from the Caribbean and Gulf of Mexico that examined the seven *Gambierdiscus* and one ribotype (undescribed species) [41]. Of these species, *G*. *excentricus* and *G*. *silvae* were more toxic than the other species occurring in the region [41]. Given interspecific variations in toxicity among *Gambierdiscus* species is high, which species are present and their relative abundances could significantly influence the potential for fish in that region to become ciguatoxic [41]. This hypothesis, however, has yet to be explicitly tested in the field. If true, it may be especially important for fish, like lionfish, which typically exhibit a high degree of site fidelity and only occasionally move distances up to 1 km in response to changes in temperature, currents, and prey availability [42]. *Gambierdiscus* abundance and species composition was not available at fish collection sites so this source of variability is unaccounted for in this study.

It should also be noted this study did not address any seasonal variation in toxicity. In the Pacific, seasonal variation in *Gambierdiscus* abundance has been shown to influence the CTX concentrations in fish [40]. Whether an equivalent seasonal variation in toxicity occurs in the Caribbean and Gulf of Mexico is unknown and needs to be addressed in future research. The relationship between CTX concentration in lionfish and the length of time they have been at a specific location is also unknown. In many sampling locations in this study lionfish have only been present for 5 to 10 years. It is possible that with longer occupation, particularly in known CFP hotspots, the percentage of lionfish containing >0.1 ppb C-CTX-1 eq. may increase.

The central aim of this study was to provide data useful in evaluating whether a viable lionfish fishery could be established as a population control strategy. Assessing the resulting data in this context is both complicated and nuanced. Top predators in the Caribbean such as amberjack (*Seriola* spp.), barracuda (*Sphyraena* spp.), grouper (*Serranidae* spp.), jacks (*Caranx* spp.), and snapper (*Lutjanus* spp.) can accumulate up to 20–50 ppb C-CTX-1 eq. and become highly ciguatoxic (Table 3). Their commercial harvesting and sales are governed by the Hazard Analysis and Critical Control Point (HACCP) protocol discussed below. The data from this study and those from the US Virgin Islands and the Lesser Antilles show lionfish from CFP prone areas can bioaccumulate CTXs [17, 18]. For example, 8 of the 55 lionfish analyzed from Saint Barthélemy, contained CTX levels that were at least 10-fold over the FDA guidance level while two of the fish tested 20- and 30-fold above [18]. The two fish containing the highest CTX levels exceeded concentrations known to cause human illness. In contrast, lionfish from areas with low overall incidence rates of CFP, such as Trinidad and Tobago, may represent little risk.

**Table 3:** Range of reported C-CTX-1 concentrations for Caribbean and Gulf of Mexico fish other than lionfish.

Study	Fish (common name)	Location	C-CTX-1 (ppb)
[51]	*Lutjanus griseus* (Grey Snapper) *Serranidae* (Grouper) *Caranx lugubris* (Black jack)	Guadeloupe	0.24–13.8
[52]	*Sphyraena barracuda* (Barracuda)	Guadeloupe	49
[53]	*Sphyraena barracuda* (Barracuda)	NW Gulf of Mexico	0.06–0.14

Given this circumstance, and with the understanding that fish are not routinely screened for CTXs, the best advice in terms of harvesting lionfish commercially is to follow the US FDA Guidance for Industry: Purchasing Reef Fish Species Associated with the Hazard of Ciguatera Fish Poisoning published November 2013 [43]. This document recommends "Because ciguatoxic endemic areas are localized, primary seafood processors should recognize and avoid purchasing fish from established and emerging areas of concern". In addition, FDA has in publication a guide [44] that identifies all hazards (e.g. ciguatera) that must be controlled through the HACCP plan. Primary processors of lionfish should refer to this document for development and implementation of a HACCP plan, which includes knowledge and written documentation of the harvest area. In brief, the best guidance available at this time for assessing the risk associated with consuming lionfish from a specific location is to rely on prior history or emerging knowledge regarding the ciguatoxicity of other fish in the area and to avoid the purchase/sale of lionfish from known high-risk areas [21, 45, 46].

Across the Pacific, where fish from CFP prone areas are consumed, it is also advised that individuals eat smaller portions of a fish and avoid multiple servings from one individual fish [21, 45, 47]. Further, fish viscera including the livers, should be avoided as they often contain much higher CTX concentrations compared to flesh [48, 49]. Associated education and outreach in CFP prone areas are also important management tools that allow consumers to make informed choices about what to eat [46, 47, 50]. A crucial part of that education is imparting the knowledge that CTXs can be cumulative over time and that eating fish with even low levels of CTXs can result in CFP [47]. These additional strategies could be considered for application in CFP prone regions in the Caribbean and Gulf of Mexico where recreationally caught lionfish are consumed.

In summary, lionfish CTX concentrations were variable depending on location. In the Caribbean and Gulf of Mexico lionfish had low incidence rates of CTX contamination above the FDA guidance level. However, in some localized areas, identified as “hot spots”, caution should be used in harvesting reef fish, including lionfish. Ideally, a monitoring program should be established to survey CTX concentrations in fish. A surveillance program could provide important data relevant to protecting human health by improving management decisions regarding CFP risks. Unfortunately, no cost effective commercial CTX tests are currently available. In the interim, any commercial harvesting of lionfish should be governed by a rigorous HACCP plan.
